# DNA-Sequence Based Typing of the *Cronobacter* Genus Using MLST, CRISPR-*cas* Array and Capsular Profiling

**DOI:** 10.3389/fmicb.2017.01875

**Published:** 2017-09-29

**Authors:** Pauline Ogrodzki, Stephen J. Forsythe

**Affiliations:** ^1^School of Science and Technology, Nottingham Trent University, Nottingham, United Kingdom; ^2^foodmicrobe.com, Nottingham, United Kingdom

**Keywords:** *Cronobacter*, genotyping, genomes, MLST, capsule, CRISPR-*cas* profiles

## Abstract

The *Cronobacter* genus is composed of seven species, within which a number of pathovars have been described. The most notable infections by *Cronobacter* spp. are of infants through the consumption of contaminated infant formula. The description of the genus has greatly improved in recent years through DNA sequencing techniques, and this has led to a robust means of identification. However some species are highly clonal and this limits the ability to discriminate between unrelated strains by some methods of genotyping. This article updates the application of three genotyping methods across the *Cronobacter* genus. The three genotyping methods were multilocus sequence typing (MLST), capsular profiling of the *K*-antigen and colanic acid (CA) biosynthesis regions, and CRISPR-*cas* array profiling. A total of 1654 MLST profiled and 286 whole genome sequenced strains, available by open access at the PubMLST *Cronobacter* database, were used this analysis. The predominance of *C. sakazakii* and *C. malonaticus* in clinical infections was confirmed. The majority of clinical strains being in the *C. sakazakii* clonal complexes (CC) 1 and 4, sequence types (ST) 8 and 12 and *C. malonaticus* ST7. The capsular profile K2:CA2, previously proposed as being strongly associated with *C. sakazakii* and *C. malonaticus* isolates from severe neonatal infections, was also found in *C. turicensis*, *C. dublinensis* and *C. universalis*. The majority of CRISPR-*cas* types across the genus was the I-E (Ecoli) type. Some strains of *C. dublinensis* and *C. muytjensii* encoded the I-F (Ypseudo) type, and others lacked the *cas* gene loci. The significance of the expanding profiling will be of benefit to researchers as well as governmental and industrial risk assessors.

## Introduction

*Cronobacter* spp. is well known with respect to outbreaks of severe infant infections (necrotizing enterocolitis and meningitis) in neonatal intensive care units. However, the majority of *Cronobacter* infections are in the adult population with various symptoms including urinary tract infections (Holy and Forsythe, 2014; Patrick et al., 2014; Alsonosi et al., 2015). The organism is also a commensal member of the human body flora. Bacterial analysis of throat swabs from over 45,000 outpatients during the period 2005–2011 recovered the organism from every age group, with the highest frequency (8.7/1000 patients sampled) from infants less than 1 year of age (Holý et al., 2014). *Cronobacter*, then known as *Enterobacter sakazakii*, was the first foodborne pathogen which the FAO-WHO aimed to control through reducing neonatal and infant exposure to contaminated reconstituted infant formula (Food and Agriculture Organization of the United Nations [FAO], 2004). This resulted in both improved microbiological criteria at point of production and revised hygienic practices of preparation (Food and Agriculture Organization of the United Nations [FAO], 2004, 2006; Food and Agriculture Organization of the United Nations [FAO] and World Health Organization [WHO], 2008). In addition, the first expert committee meeting made various recommendations, including the need for an internationally validated detection and molecular typing methods for *Cronobacter* spp. and other relevant microorganisms (Food and Agriculture Organization of the United Nations [FAO], 2004).

This paper considers how, since 2004, these recommendations have been met through molecular typing methods based on the application of NGS over conventional methods. Reviews of wider aspects of *Cronobacter*, such as environmental fitness and virulence traits, have been recently published and therefore will not be considered in detail here (Forsythe et al., 2014; Almajed and Forsythe, 2016).

Powdered infant formula (PIF) has been the main vector associated with *Cronobacter* spp. and therefore has been the focus for the reduction in neonatal infections. Consequently, the Food and Agriculture Organization of the United Nations [FAO] (2004) encouraged the establishment of detection and molecular typing schemes which could be used to monitor sources of *Cronobacter* in PIF. An initial challenge was the differentiation of *Cronobacter* spp. (then known as *Enterobacter sakazakii*) from closely related organisms which could be co-recovered from infant formula and its ingredients, i.e., *Franconibacter*, *Siccibacter* and *Enterobacter* spp. (Stephan et al., 2014). Therefore an accurate taxonomic description of *Cronobacter* spp. was necessary for both reliable detection method development and for appropriate regulatory control. It should also be noted that other sources have been reported including the water used for reconstitution of PIF reconstitution, and also enteral feeding tubes (Hurrell et al., 2009b; Broge and Lee, 2013; Hariri et al., 2013; Ravisankar et al., 2014).

Unfortunately, some *Cronobacter* detection methods have been based on poorly characterized, even misidentified, strains (Jackson and Forsythe, 2016). The various *Cronobacter* species were initially defined according to the 16 *Enterobacter sakazakii* biotypes, however some of the biotype index strains were assigned the wrong *Cronobacter* species and this has limited further development of accurate phenotypic methods for *Cronobacter* identification (Iversen et al., 2008; Baldwin et al., 2009; Joseph et al., 2013a; Jackson and Forsythe, 2016). Additionally, the earlier reliance on phenotyping methods led to a number of mistaken identifications in the literature (Caubilla-Barron et al., 2007; Townsend S. et al., 2008; Blažková et al., 2015; Jackson et al., 2015a; Ogrodzki and Forsythe, 2015).

Such misidentifications can cause further confusion for risk management and the control of infection, as well as misinformation on current issues such as carriage of antibiotic resistance. Therefore reliable and robust means of identifying and typing *Cronobacter* isolates are required and should be internationally accessible.

Although 16S rDNA sequence analysis is generally applicable for bacterial species identification, it is not a reliable method for members of the *Cronobacter* genus as it is unable to reliably differentiate between the two species *C. sakazakii* and *C. malonaticus* (Iversen et al., 2008; Baldwin et al., 2009). In order to overcome this limitation, Joseph et al. (2012b) used representative strains across the genus which had been selected using multilocus sequence analysis (MLSA) of 7 housekeeping genes; ATP synthase b chain (*atpD*), elongation factor G (*fusA*), glutaminyl tRNA synthetase (*glnS*), glutamate synthase large subunit (*gltB*), DNA gyrase subunit B (*gyrB*), translation initiation factor IF-2 (*infB*) and phosphoenolpyruvate synthase A (*ppsA*). This DNA-sequence based approach overcame the preconceived grouping of strains based on phenotyping, and supported the recognition of two further *Cronobacter* species; *C. universalis* and *C. condimenti* (Joseph et al., 2012a).

There are a number of centralized MLST databases which are internationally available covering bacteria and fungi with standardized allele profile determination programs. The three major bacterial MLST databases are those at the Institute of Pasteur^1^, and the universities of Warwick^2^, and Oxford^3^. Initially MLST used laboratory-based sequencing of individual genes, but nowadays it is more reliant on *in silico* analysis of whole genomes. The analysis of uploaded *Cronobacter* genome sequences is through the ‘Bacterial Isolate Genomes Sequence Database’ (BIGSdb) facility^4^. Furthermore the inclusion of whole genomes has enabled the *Cronobacter* MLST database to be expanded to ribosomal-MLST (r-MLST; 53 genes) and core genome MLST (cg-MLST; 1836 genes) analysis to provide greater resolution between strains and deeper bacterial population studies (Maiden et al., 2013; Forsythe et al., 2014; Jolley and Maiden, 2014). Phylogenetic comparison of 7-loci MLST, rMLST, cg-MLST and whole genome analysis has confirmed the reliability and robustness of the 7-loci MLST scheme for speciation and subtyping within the *Cronobacter* genus as well as differentiation from related genera. Additionally, *fusA* allele phylogeny corresponds to whole genome phylogeny and can be used for initial speciation of *Cronobacter* isolates (Joseph et al., 2012c; Forsythe et al., 2014).

The use of MLST, based on NGS, has therefore supported defining the *Cronobacter* genus. The genus is within the family *Enterobacteriaceae*, with the nearest relatives being the *Franconibacter* and *Siccibacter* genera, as well as the more familiar genera of *Enterobacter*, *Citrobacter* and *Pantoea* (Stephan et al., 2014; Jackson et al., 2015b). According to the MLST phylogenetic analysis, the genus split from its nearest ancestor in the *Enterobacteriaceae* family *ca.* 45–68 million years ago, at the same time as the *Salmonella* genus diverged into its species and subspecies (McQuiston et al., 2008; Joseph and Forsythe, 2012).

Using *in vitro* virulence studies, it has been shown that *Cronobacter* spp. can invade intestinal and brain cells, as well as persist and even replicate in macrophages (Townsend et al., 2007; Townsend S.M. et al., 2008; Almajed and Forsythe, 2016). The most reported *Cronobacter* species in clinical cases are *C. sakazakii* and *C. malonaticus* in infant and adults, respectively (Forsythe et al., 2014; Alsonosi et al., 2015). A number of virulence traits have been proposed which may account for these clinical differences. Nevertheless, there have been insufficient large scale comparative whole genome studies to establish their contribution to severe clinical presentations. Of particular interest has been the predominance of *C. sakazakii* in neonatal infections, especially CC4 and ST12. However, studies have not identified traits unique to specific *Cronobacter* pathovars (Kucerova et al., 2010, 2011; Joseph and Forsythe, 2011; Joseph et al., 2012b; Masood et al., 2015). This could be due to unrecognized synergy between different virulence traits and environmental fitness traits leading to increased neonatal exposure and severity of infection (Hariri et al., 2013).

*Cronobacter sakazakii* strains are able to use sialic acid as a carbon source for growth (Joseph et al., 2013b). This could be a highly significant host-adaptation trait since sialic acid is found in breast milk, mucin and gangliosides (Almagro-Moreno and Boyd, 2009). Laboratory studies have shown that *C. sakazakii* is able to grow on both sialic acid and ganglioside (GM1) as a sole carbon source (Joseph et al., 2013b) and could enable the degradation of intestinal mucin, facilitating access to intestinal cells. Due to its association with brain development, sialic acid is an ingredient in PIF and therefore an additional *C. sakazakii* growth substrate following rehydration. In contrast, unlike other *Cronobacter* species, *C. sakazakii* strains are unable to grow on malonic acid. This organic acid is found in plants and the ability to utilize it is regarded as reflecting the initial plant-association of the *Cronobacter* genus (Schmid et al., 2009). The adaptation of *C. sakazakii* to a new ecosystem, with the subsequent loss of malonic acid utilization and gain in sialic acid utilization might therefore contribute to its pathogenicity toward neonates (Joseph et al., 2013b). The mechanisms and routes of exposure by which *C. malonaticus* infect adults more than infants are as yet unknown. In the *Cronobacter* genera, a total of 10 fimbrial families have been identified. *C. sakazakii* lack curli fimbriae, but are unique in encoding for β–fimbriae (Joseph et al., 2012b). The clinical significance of this has not been investigated, but might contribute to the greater predominance of *C. sakazakii* in infant infections through attachment to host cells. A range of other virulence traits have been reported. These include *Cronobacter* plasminogen activator (cpa) (Franco et al., 2011), iron utilization (Grim et al., 2012), outer membrane vesicle release causing host cell cytopathogenicity (Alzahrani et al., 2015; Kothary et al., 2017), as well as heavy metal resistance traits (copper, silver, zinc, tellurite) (Joseph et al., 2012b; Chaturvedi et al., 2015).

*Cronobacter* spp. produce capsular material composed of the *O* and *K*-antigens, colanic acid, Enterobacteriacae common antigen, and cellulose (Ogrodzki and Forsythe, 2015). The capsular material is composed of water-saturated, high molecular weight polysaccharides. It plays a role in virulence by enabling organisms to evade host response mechanisms (serum resistance and phagocytosis), as well as facilitating biofilm formation and desiccation survival (Willis and Whitfield, 2013). *Cronobacter* biofilms have been found in hospitals on equipment used to prepare infant formula, feeding bottles, and neonatal enteral feeding tubes (Iversen and Forsythe, 2003; Iversen et al., 2004; Kim et al., 2006; Hurrell et al., 2009a,b; Holy and Forsythe, 2014; Jackson et al., 2015a). The biofilms on enteral feeding tubes could act as loci for neonatal infection (Hurrell et al., 2009a,b). Under some growth conditions, the organism produces excessive capsule material which may protect the organism from disinfectants in food production environments, and enable long-term persistence under desiccation conditions, such as in PIF for over 2 years (Caubilla-Barron and Forsythe, 2007). It is therefore plausible that the strong association between *C. sakazakii* CC4 and neonatal meningitis is due to its environmental fitness of desiccation survival, resulting in its persistence in PIF and the environment of PIF manufacturing plants (Muller et al., 2013; Power et al., 2013; Sonbol et al., 2013; Fei et al., 2015, 2017). Therefore the greater incidence of *C. sakazakii* CC4 in PIF could lead to an increased risk of exposure and incidence of infection rather than being due to the organism encoding for specific pathogenicity traits.

A capsular profiling scheme for *Cronobacter* based on the *K*-antigen and colanic acid (CA) biosynthesis encoding genes has been proposed (Ogrodzki and Forsythe, 2015). This scheme was based on the analysis of 104 *Cronobacter* genomes and revealed that strains of *C. sakazakii* and *C. malonaticus* isolated from cases with the most severe neonatal clinical presentations of invasive meningitis and necrotizing enterocolitis (NEC), had a definable capsular profile which differed from strains associated with less severe clinical cases. They reported that all (*n* = 54) strains of *C. sakazakii* CC4 and ST12 strains known for being associated with severe neonatal infections of meningitis and NEC, had the capsular profile K2:CA2 (Joseph and Forsythe, 2011, 2012; Joseph et al., 2012c; Hariri et al., 2013; Forsythe et al., 2014). Of particular interest was that this particular capsule profile was also found in the isolates of the few *C. sakazakii* non-CC4 cerebral spinal fluid (CSF) cases, including a single *C. malonaticus* fatal case (Ogrodzki and Forsythe, 2015). Also of interest was that two *C. turicensis* strains encoding for sialic acid utilization also had the capsular profile K2:CA2. Since the earlier study by Ogrodzki and Forsythe (2015), a large number of *Cronobacter* genomes have become available and therefore a wider evaluation of capsular profiling across the genus is now feasible.

The establishment of internationally standardized molecular typing methods applicable across the *Cronobacter* genus is necessary given the severe outcomes of infections in neonates and the attributed link to contaminated PIF on the international market (Food and Agriculture Organization of the United Nations [FAO], 2004, 2006; Food and Agriculture Organization of the United Nations [FAO] and World Health Organization [WHO], 2008). Although it is generally possible to differentiate *Cronobacter* species by biochemical profiling, molecular methods are increasingly used as a more rapid and reliable tool to study bacterial genomic diversity and to track sources of infection. Since *Cronobacter* is ubiquitous, such typing schemes are applicable for both epidemiological and environmental investigations. For epidemiological analysis (i.e., tracing source and dissemination during an outbreak), PFGE with two restriction enzymes (*Xba*1 and *Spe*1) has in the past been the most common method (van Acker et al., 2001; Himelright et al., 2002). The method is limited, however, as not all *Cronobacter* strains can be typed due to intrinsic DNase activity, non-identical strains can give the same PFGE profile and the method does not give the relationship between strains (Centers for Disease Control and Prevention [CDC], 2010; Alsonosi et al., 2015). Due to these limitations of PFGE, the Centers for Disease Control and Prevention (CDC) is transitioning to using whole genome sequencing as the basis for PulseNet surveillance (Carleton and Gerner-Smidt, 2016; Nadon et al., 2017).

A number of typing methods for *Cronobacter* have been published, in particular PCR-probe *O*-antigen serotyping (Mullane et al., 2008; Jarvis et al., 2011; Sun et al., 2011). Initially 7 serotypes were defined for *C. sakazakii* and 2 in *C. malonaticus*. However, some strains of *C. malonaticus* were mis-identified as *C. sakazakii* by Sun et al. (2011) and were assigned *C. sakazakii* serotypes O:5 and O:6. Consequently, Blažková et al. (2015) proposed the O-antigen scheme should be revised with additional recognition of 7 new and 2 re-assigned serotypes. Chemical analysis of the *O*-polysaccharide units from many strains has confirmed the molecular *Cronobacter* serotypes. However three structures have been determined for *C. sakazakii* O:2 strains (Arbatsky et al., 2010; Czerwicka et al., 2010; Maclean et al., 2010). This is probably due to variants in the *O*-antigen genes which occur outside the target region of the PCR primers.

The PCR-probe O-serotyping has been superseded by allele profiling of *gnd* and *galF* (encoding 6-phosphogluconate dehydrogenase and UTP-glucose-1-phosphate uridylyltransferase subunits, respectively) (Ogrodzki and Forsythe, 2015). This DNA-sequence based approach is a more reliable and expansive method for *O*-antigen determination within *Cronobacter*. It has increased the definable number of serotypes in the *Cronobacter* genus from 18 to 34 (Ogrodzki and Forsythe, 2015).

MLST analysis has not only been able to support taxonomic revisions, but can differentiate strains to a greater degree than other methods; >500 defined STs compared with 34 for O-serotyping. It has also revealed a remarkably strong clonal nature in the *Cronobacter* genus, in particular within the clinically relevant *C. sakazakii* and *C. malonaticus* species (Baldwin et al., 2009; Joseph et al., 2012c). This has subsequently led to the recognition of particular pathovars (Joseph and Forsythe, 2011; Hariri et al., 2013; Sonbol et al., 2013; Forsythe et al., 2014). *C. sakazakii* CC4 is a DNA sequence defined evolutionary lineage which is especially associated with neonatal meningitis. *C. sakazakii* ST12 is associated with cases of NEC and not neonatal meningitis or septicaemia. *C. sakazakii* CC1 strains are primarily isolated from infant formula and clinical sources, whereas *C. sakazakii* ST8 are isolated from clinical and non-formula food sources. *C. malonaticus* adult infections are almost exclusively CC7.

While this clonal recognition is useful for the identification of *Cronobacter* pathovars, it is counter-productive for microbial source tracking as unrelated strains will occur in the same ST. This may explain the observation that the same PFGE pulsotype can be obtained for unrelated clinical *C. sakazakii* strains (Forsythe et al., 2014; Alsonosi et al., 2015). In order to address this issue, two further typing methods have been applied to discriminate strains within a given ST; capsule profiling and ‘CRISPRs’ and CRISPR-associated genes (*cas*) protein-coding genes (CRISPR-*cas*) array profiling. These were chosen as two independent typing tools as capsular genes do not follow phylogeny, and the CRISPR-*cas* array reflects the exposure of strains to phages and plasmids (Ogrodzki and Forsythe, 2015; Zeng et al., 2017).

There are a number of different CRISPR-*cas* systems, often named according to their first identification organism, i.e., *E. coli* (type I-E) and *Yersinia pseudotuberculosis* (type I-F) (Makarova et al., 2015). In general, CRISPR-*cas* systems may have up to three sections (a) *cas* genes (b) an AT-rich leader sequence upstream of the array and (c) a CRISPR array, composed of short (∼24–48 nucleotide) direct repeat sequences separated by similarly sized, unique spacers which are usually derived from mobile genetic elements such as bacteriophages and plasmids (Grissa et al., 2007b; Makarova et al., 2015). CRISPR-*cas* systems may have a number of roles such as adaptive immunity to phages and plasmids, as well as bacterial virulence and stress response (Barrangou et al., 2007; Shariat and Dudley, 2014).

Many applications have been identified for the CRISPR-*cas* system such as gene editing, evolutionary and phylogenetic studies, as well as genotyping for epidemiological investigations (Fricke et al., 2011; Makarova et al., 2015). CRISPR arrays may differ between closely related strains due to their different exposure histories to phages and plasmids, leading to differences in their spacer acquisitions. Therefore these loci can be used for molecular subtyping, offering greater discrimination between strains than MLST and PFGE, especially useful for highly clonal species, such as *C. sakazakii.*

This paper considers the diversity of *Cronobacter* across the genus according to 7-loci MLST, the association between capsule profile of the K-antigen and colanic acid biosynthesis genes with clinical presentation, and compares CRISPR-*cas* array profiling between the highly clonal *Cronobacter* species *C. sakazakii* with less clonal species *C. dublinensis*. Of particular relevance is the availability of over twice the number of genomes (280 compared with 104) since previous publications on capsule and CRISPR-*cas* array profiling (Forsythe et al., 2014; Ogrodzki and Forsythe, 2015, 2016).

## Materials and Methods

### Strains Used in This Study

A total of 1654 STd strains and 275 genomes were included in this study (**Table 1**). Strains from patients less than 1 year in age were defined as infant in origin, those from patients ages 1–15 were termed child, and those above 15 were regarded as adult isolates. Additional metadata can be obtained from the open access *Cronobacter* PubMLST database; http://pubmlst.org/cronobacter/.

**Table 1:** Summary of *Cronobacter* isolates in the *Cronobacter* PubMLST database.

						Source				
Species	Number of strains (%)	Number of STs^a^	Number of genomes	Earliest isolate	Countries	Clinical	Infant formula	Food and ingredients	Environmental	Other
*C. sakazakii*	1126 (68.1)	236	155	1950	32	14.5^b^	21.5	43.4	17.6	3.0
*C. malonaticus*	222 (13.4)	94	55	1973	17	26.4	13.7	44.8	8.5	6.6
*C. dublinensis*	155 (9.4)	107	31	1956	12	3.0	5.3	63.9	25.6	2.3
*C. turicensis*	76 (4.6)	46	14	1970	13	8.0	4.0	52.0	31.4	6.7
*C. muytjensii*	57 (3.4)	28	10	1988	12	1.8	10.7	53.6	1.8	32.1
*C. universalis*	16 (1.0)	9	8	1956	6	5.6	0.0	55.6	22.2	16.7
*C. condimenti*	2 (0.1)	1	2	2010	1	0.0	0.0	100	0	0
Total	1654	521	275		36	14.2^c^	17.5	46.4	17.2	4.7

### Seven Loci MLST Analysis

As per Forsythe et al. (2014), DNA sequences collated at http://pubmlst.org/cronobacter/ were investigated. Concatenated sequences of seven loci from 521 STs were downloaded in FASTA format using the Export/Sequences option. These sequences were aligned in MEGA version 6.05 using the ClustalW algorithm (Tamura et al., 2013). The final alignment spanned 3036 bp and was analyzed using the default pipeline in SplitsTree4 (UncorrectedP to calculate distances and NeighborNet to build the network) (Kloepper and Huson, 2008).

### Goeburst Analysis

Phyloviz version 2.0 tool was used with goeBURST Full MST algorithm. Level 3 (TLV) was chosen which represents the Locus Variant level and removes of all the links greater than the number selected (Nascimento et al., 2017).

### Bacterial Genome Analysis

As per Ogrodzki and Forsythe (2015, 2016). A total of 275 genomes were analyzed for this study, which were the total number of genomes available (March 2017). Of particular interest were 41 whole genome sequenced isolates were chosen as representatives of *C. dublinensis* and *C. muytjensii*. They were geographically dispersed over 12 countries and temporally spread over 50 years (**Table 1**). Additional metadata can be obtain from the open access *Cronobacter* PubMLST database; http://pubmlst.org/cronobacter/.

### Defining CRISPR-*cas* Arrays

CrisprFinder tool was used to identify number of Crispr arrays, including the number and length of DR and spacers sequences; http://crispr.i2bc.paris-saclay.fr/ (Grissa et al., 2007a)

### Phylogenetic Analysis

As per Ogrodzki and Forsythe (2015, 2016). DNA sequences were carefully curated prior to and after alignment and phylogenetic analyses in order to maximize the quality of the results using the satisfactory default parameters for the latter analyses. DNA sequences were aligned in MEGA version 6.05 using the ClustalW algorithm (Tamura et al., 2013) set to default parameters settings. The phylogenetic trees were generated using the Maximum Likelihood (ML) method based on the Tamura-Nei model with the additional parameters set to default settings. All phylogenetic trees are drawn to scale with branch lengths measured in the number of substitutions per site.

### DNA Sequences

As per Ogrodzki and Forsythe (2016). Whole genome DNA sequences collated at http://pubmlst.org/cronobacter/ were investigated. *In silico* analyses were carried out using search options, such as BLAST, on the *Cronobacter* PubMLST portal accessible at: http://pubmlst.org/perl/bigsdb/bigsdb.pl?db=pubmlst_cronobacter_isolates.

### DNA Annotation and Visualization Tools

As per Ogrodzki and Forsythe (2015, 2016). Nucleotide sequences were extracted from the corresponding genome assemblies in the *Cronobacter* PubMLST database.

Bacterial DNA sequences were investigated using the genome browser and annotation tool Artemis (Carver et al., 2012).

## Results

### *Cronobacter* Diversity and Source According to MLST

The MLST scheme for *Cronobacter* open access database^5^ was used as the source of strain sequences and metadata. At the time of writing this database contained over 1654 strain profiles, including >270 whole genomes, along with associated metadata such as source, date of isolation, and clinical presentations (**Table 1**). The strains had been collected from various sources (clinical, food and environmental) and countries over a 60 year period. Since the database also contains the metadata for over 1600 isolates, an informed understanding of the diversity and sources of the *Cronobacter* genus can be obtained. The database can also be used for the retrospective analysis of strains from earlier studies.

Investigating 1654 isolate entries in the *Cronobacter* MLST database revealed the temporal, geographic and source diversity of the organism (**Table 1**). *Cronobacter* strains have been isolated from 36 countries, and are from clinical (14.2%), infant formula (17.5%), food and food ingredients (46.4%), and environmental (17.2%). The earliest isolate (*C. sakazakii* NCIMB 8282) was isolated from dried milk powder in 1950.

**FIGURE 1:**
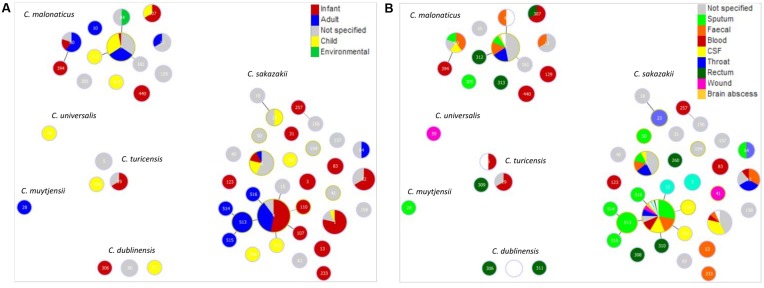
Genetic relationship of clinical *Cronobacter* isolates according to **(A)** patient age along with clinically relevant environment and **(B)** isolation site. The minimum spanning tree was constructed using goeBurst analysis of 53 STs from 234 strains. The size of the circle is proportional to the number of strains.

Given the primary interest in *Cronobacter* is due to clinical infections, Goeburst analysis was used to visualize the diversity of clinical isolates according to their ST, patient age and site of isolation; **Figure 1**. The majority of strains were from *C. sakazakii* (68.1%) and *C. malonaticus* (13.4%). Out of a total of 236 defined STs for *C. sakazakii*, the major STs recovered from clinical sources were STs 1, 4, 8, and 12. ST4 was the most numerous (208 strains out of 1126) ST in the database. The ST4 isolates were primarily from infants (53%), and secondly adults (37%); **Figure 1A**. Other *C. sakazakii* STs of note were STs 1, 8, and 12 which were primarily composed of infant isolates; 79% (*n* = 14), 14% (*n* = 14) and 67% (*n* = 9), respectively. As shown in **Figure 1B**, the site of isolation for *C. sakazakii* ST4 was most frequently sputum (31%), CSF (16%) and feces (17%). Isolates of *C. sakazakii* ST8 were also from a range of sources (throat, fecal, sputum, CSF), whereas ST1 were from fewer sites; primarily CSF (36%) for ST1, and feces and throat (33% each) for ST12. *C. malonaticus* ST7 was the major (29% n = 199) *C. malonaticus* ST. Sixteen *C. malonaticus* ST7 strains had detailed clinical information. These had been recovered from throat, feces, and sputum samples; **Figure 1B**. The remaining 5 *Cronobacter* species together composed 18.5% of the database, and contained only 13 clinical isolates in total (**Table 1**).

### Capsule Profiling and Distribution of Sialic Acid Utilization Genes across the *Cronobacter* Genus

**Table 2** summarizes the occurrence of K-antigen and colanic acid biosynthesis genes across 275 whole genome strains from the *Cronobacter* genus. The K2:CA2 profile, previously reported to be associated with neonatal meningitis cases, was primarily found in clinical isolates (*n* = 59/275). Most (*n* = 84/103) were *C. sakazakii* strains, in 16 STs. The K2:CA2 profile was found in 10 *C. malonaticus* strains of ST60 and ST307, of which 7 were clinical in origin. Previously only one *C. malonaticus* reported case of severe meningitis in an infant had been documented (Ogrodzki and Forsythe, 2015). This was a fatal meningitis case of an infant born with an EGA of 32 weeks in America (2011). The culture sequenced was a blood isolate, and was presumed to be the infectious organism of the brain. This single case was proposed by Ogrodzki and Forsythe (2015) as possible outlier evidence of K2:CA2 link to meningitis. In this paper a second *C. malonaticus* K2:CA2 isolate (ID 1494) is reported. This strain was isolated in China (2014) from the CSF of an infant born with an EGA of 36 weeks, who was fed fortified breast milk and developed clinical symptoms of meningitis on day 11. Of the remaining *C. malonaticus* strains with the K2:CA2 profile, three were from adults aged 26–82 years (no clinical symptoms available). There were no clinical details for the remaining 2 *C. malonaticus* strains. The K2:CA2 profile was also found in a total of 9 *C. turicensis*, *C. dublinensis* and *C. universalis* strains, none of which were clinical in origin. The second most frequent capsule profile was K1:CA1 (*n* = 89/275), of which 31 were clinical in origin but did not correspond to any specific clinical presentation (**Table 2**). This profile was not found in *C. condimenti*, *C. universalis*, or *C. turicensis*.

**Table 2:** Distribution of capsule profile (*K*-antigen and colanic acid genes) and presence of sialic acid utilization genes across the *Cronobacter* genus.

					Source		
Capsule Capsule profile	Species	Sequence type (ST)	Number of strains	Number of strains encoding for sialic acid utilization genes^a^	Clinical	Non-clinical	Unknown
		CC4^b^, 12, 13, 31, 40,					
K2:CA2	*C. sakazakii*	184, 3, 233, 136, 83	84	84	52	32	0
	*C. malonaticus*	60, 307	10	0	7	2	1
	*C. turicensis*	35, 342	3	3	0	1	1
	*C. turicensis*	24, 252, 387	4	0	0	5	0
	*C. dublinensis*	301	1	0	0	0	1
	*C. universalis*	137	1	0	0	1	0
	Subtotal (24)^c^		103	87	59	41	3
	*C. sakazakii, C. malonaticus*,						
K1:CA1	*C. muytjensii, C. dublinensis* (19)		89	44	31	56	2
K2:CA1	*C. sakazakii, C. universalis* (13)		15	14	3	10	2
K1:CA2	All *Cronobacter* species (46)		68	17	9	40	15
	Subtotal (60)		172	75^d^	43	106	23
	Total		275	162	102	147	26

All *C. sakazakii* strains encoded for the sialic acid utilization genes (*yhch-nanKTAR*); **Table 2**. These genes were not found in any other species, except 7 (out of 14) *C. turicensis* strains, of which 3 had the K2:CA2 capsular profile and the remainder had the profile K1:CA2. The *C. turicensis* strains encoding for sialic acid utilization were in five different STs; 24, 35, 252, 342, and 387. The sialic acid utilization genes were not found in any other *Cronobacter* species.

### CRISPR-*cas* Operon Architecture

**Table 3** presents the detailed CRISPR-*cas* profiling of 100 isolates. The previously published data for the four clinically significant *C. sakazakii* pathovars (CC1, CC4, ST8 and ST12) are shown for comparative purposes with *C. dublinensis* and *C. muytjensii*. The latter two species were chosen for detailed analysis as they had not been the focus of earlier CRISPR-*cas* array studies (Ogrodzki and Forsythe, 2016; Zeng et al., 2017). *C. muytjensii* and *C. dublinensis* do not show such strong clonality compared with *C. sakazakii* and *C. malonaticus* resulting in the higher proportion of unique STs for the number of strains in the *Cronobacter* database (**Table 1**). It was therefore predicted that the diversity of the CRISPR-*cas* profiles might be greater in *C. dublinensis* and *C. muytjensii.* The genomes of *C. dublinensis* and *C. muytjensii* strains studied were widely temporally (58 years) and globally (11 countries) distributed in their origin and therefore representative of the diversity of the two species.

**Table 3:** CRISPR-*cas* operon structure and array profile variation in *Cronobacter* spp.

*Cronobacter species*	Sequence type or clonal complex	Number of strains	Operon structure type^a^	Maximum number of CRISPR arrays per strain	Maximum number of spacers per strain	Reference
	76, 77, 79, 80, 106, 213,				
*C. dublinensis*	301, 341, 346, 388, 389	13	I-E^b^	4	66	This study
*C. dublinensis*	80, 95, 409	4	I-F^c^	5	47	This study
*C. dublinensis*	70, 74, 389	3	No *cas* genes	1	14	This study
*C. muytjensii*	407, 411	2	I-E	2	20	This study
*C. muytjensii*	81	5	I-F	3	45	This study
*C. muytjensii*	294, 347, 403	3	No *cas* genes	1	15	This study
*C. sakazakii*	CC4	25	I-E	2	23	[1]
*C. sakazakii*	CC1	29	I-E	3	30	[1]
*C. sakazakii*	12	8	I-E	3	11	[1]
*C. sakazakii*	8	8	I-E	4	18	[1]

Twenty genomes of source distributed and ST diverse *C. dublinensis* strains were analyzed. Thirteen genomes revealed CRISPR-*cas* arrays with the same general I-E structure, also known as Ecoli or CASS2 type, due to the presence of *cas3, cse1, cse2, cas7, cas5, cas6e, cas1*, and *cas2.* There was a maximum of 4 CRISPR arrays per strain, with up to 66 spacers. Four genomes (ST 80, 95 and 409) contained I-F (Ypest or CASS3) type CRISPR-*cas* structure, encoding for *cas1, cas2-3, csy1, csy2, csy3*, and *cas6f.* The strains had up to 5 CRISPR arrays per strains, and up to 47 spacers. As shown in **Table 3**, the two strains from ST80 differed in their operon structure; one being I-E and the other I-F. Three genomes from STs 70, 74 and 389 did not encode for any *cas* genes, yet did encode for one CRISPR array composed of 14 spacers.

Ten *C. muytjensii* genomes which were diverse with respect to their temporal, source and ST were analyzed. Five ST81 strains contained the I-F (Ypest or CASS3) type CRISPR-*cas* structure, with a maximum of 5 CRISPR arrays, and 45 spacers per strain. Two genomes (ST 407 and ST411) revealed CRISPR-*cas* arrays with the general I-E (Ecoli or CASS2) type CRISPR-*cas* structure. There was a maximum of 2 CRISPR arrays per strain, with up to 20 spacers. Three genomes from STs 294, 347, and 403 did not encode for any *cas* genes, but did encode for one CRISPR array composed of 15 spacers.

For completeness of the relative comparative analysis, the CRISPR-*cas* profiles of the species types strains of the remaining three *Cronobacter* species, *C turicensis*, *C. universalis*, and *C. condimenti*, were determined and included in the analysis (**Table 3**). The type strains for *C. universalis* and *C. condimenti* did not contain any *cas* genes.

**FIGURE 2:**
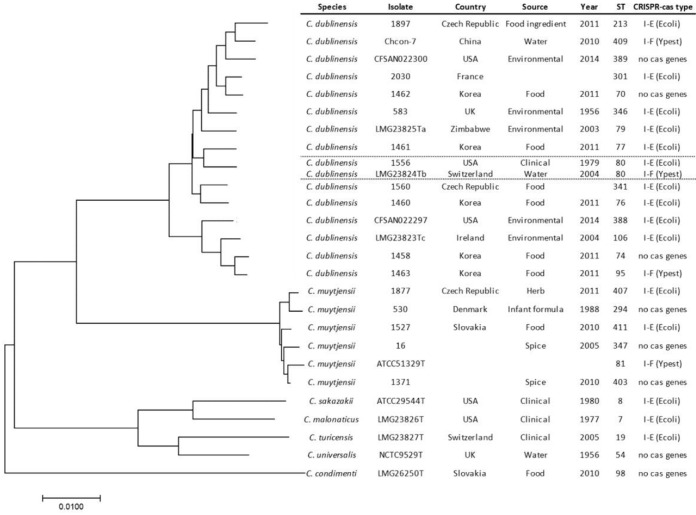
MLST phylogenetic tree of *C. dublinensis* (16 STs) and *C. muytjensii* (6 STs) and species type strains of *C. sakazakii*, *C. malonaticus*, *C. turicensis*, *C. universalis*, and *C. condimenti.* The phylogenetic tree is drawn to scale with branch lengths measured in the number of substitutions per site. Scale bar represents nucleotide substitutions.

In order to investigate any phylogenetic relationship with the CRISPR-*cas* type across the *C. dublinensis* and *C. muytjensii* species, phylogenetic analysis of the *Cronobacter* genus based on 7-loci MLST is shown in **Figure 2**. The *C. dublinensis* species cluster is very diverse, compared with *C. muytjensii*, yet can be divided into two subgroups. The smaller subgroup contains the *C. dublinensis* subsp. *dublinensis* type strain, whereas *C. dublinensis* subsp. *lactaridii* and *C. dublinensis* subsp. *lausanensis* are in the larger cluster. There was no phylogenetic association between the distribution of the I-E and I-F type CRISPR-*cas* operon structures across *C. dublinensis* and *C. muytjensis*, or across the whole genus. In addition, the two *C. dublinensis* ST80 strains (isolated in 1979 and 2004 in United States and Switzerland respectively) differed in their CRISPR-*cas* type (**Table 3**).

## Discussion

### Diversity of *Cronobacter* Species According to 7-Loci MLST

Due to initial concerns of the association between *Cronobacter* in PIF and infant infection, the organism became the focused attention for new identification and typing schemes. The primary goals being to reduce the risk of neonate exposure to the organism, and facilitate monitoring for the organism in PIF ingredients and associated manufacturing environments. The differentiation of the organism from closely related genera, such as *Franconibacter* and *Siccibacter*, has been achieved through a series of taxonomic revisions describing the *Cronobacter* genus (Stephan et al., 2014; Jackson et al., 2015b). While the considerable diversity within the *Cronobacter* genus has been recognized through DNA-sequence based typing schemes. These achievements have been through the application of NGS, and the centralization of the sequences in an open access database repository along with associated metadata. Therefore meeting the recommendations of the FAO-WHO expert committee as detailed in the Introduction (Food and Agriculture Organization of the United Nations [FAO], 2004).

The curated open access *Cronobacter* PubMLST database has been established at the University of Oxford for the genus along with associated metadata for each deposited strain; http://pubmlst.org/cronobacter/ (Forsythe et al., 2014). This database has enabled the recognition of identifiable *Cronobacter* clonal lineages within the genus as pathogenic variants, whereas others are primarily commensal organisms of the environment. The original 7-loci MLST scheme is congruent with both 53-loci rMLST and 1865-loci cg-MLST as well as whole genome phylogeny [Forsythe et al., 2014].

Although the *Cronobacter* PubMLST database cannot be used for direct enumeration purposes, the submission of new strains and sequences to the database will reflect the diversity of the genus with respect to global distribution and source variation. Additionally, the choice of strains genotyped and genome sequenced by different research groups will have been for different rationales, i.e., clinical significance, representative of species, or diversity study. Consequently, outbreak investigations may result in repeated isolation of indistinguishable strains from the same location being deposited in the database. Nevertheless this study of 1654 strains and 275 genomes is the most comprehensive to date and will serve as a reference for further specific studies and can be compared with the earlier analysis of 1000 strains (Forsythe et al., 2014).

All *Cronobacter* species have been isolated from food and food ingredients, and this source comprises 46.4% of the total profiled strains in the database (**Table 1**). These are primarily from starch-based ingredients, cake mixes, packet soup, salads, flavored teas as well as herbs and spices. Another source for *Cronobacter* spp. given in the database is the environment (17.2% entries) which included the manufacturing environment as well as the natural environment with isolates from water and soil. Despite reconstitution water being reported as the source of one reported serious *C. sakazakii* infection, water as a source of the bacterium has not received much attention (Hariri et al., 2013; Liu et al., 2013). The remaining strains in the database were clinical (14.2%) and PIF (17.5%) in origin. The major species were *C. sakazakii* and *C. malonaticus* (68.1 and 13.4%, respectively), which have been isolated from 32 countries, the earliest isolate being from dried milk powder in 1950 and the genome of which has been published (Masood et al., 2013).

The designation ‘clinical’ needs to be used with caution as it can mean a plethora of sources; symptomatic and asymptomatic patients, from normally sterile sites and non-sterile sites. For example, some strains of *C. sakazakii* isolated from neonatal feeding tubes are designated as ‘clinical’ as this was deemed more appropriate than ‘environmental,’ even though there were no associated infections reported. Consequently we have focused on isolates of clinical significance, such as those from CSF and blood with respect to proposing specific pathovars in the genus.

The four major pathovars in the *Cronobacter* genus are (i) *C. sakazakii* CC4, which is more predominantly associated with neonatal meningitis, (ii) *C. sakazakii* ST12 with neonatal necrotizing enterocolitis, (iii) *C. sakazakii* CC1 strains are primarily isolates from infant formula and clinical sources, and (iv) *C. sakazakii* ST8 were isolated from clinical and non-formula food sources (Forsythe et al., 2014). The reason for the predominance of these pathovars could be due to their persistence during the manufacture and storage of infant formula which would result in increased neonatal exposure, as well as encoding for virulence genes (Joseph and Forsythe, 2011).

The pathovar *C. sakazakii* CC4 comprises single (SLV), double (DLV) and triple (TLV) loci variants of *C. sakazakii* ST4. This clonal complex was the major one recovered from clinical sources (208/1126). They were primarily from infants (53%) as well as adults (37%) (**Figure 1A**). The reason for *C. sakazakii* CC4 association with neonatal meningitis is uncertain since no unique virulence traits had been determined in this ST (Masood et al., 2015). However, it could be linked to environmental fitness, in particular desiccation resistance accounting for the reported recovery of *C. sakazakii* CC4 strains from PIF and infant formula manufacturing plants in China, Ireland, Switzerland, Australia, and Germany (Craven et al., 2010; Reich et al., 2010; Jacobs et al., 2011; Sonbol et al., 2013; Yan et al., 2013). Fei et al. (2015) reported that, according to PFGE, environmental isolates of *C. sakazakii* CC4 from a PIF manufacturing plant in Switzerland were indistinguishable from those in the finished product. Therefore *C. sakazakii* CC4 may be an environmentally persistent clonal complex which due to increased neonatal exposure results in infant infections. Other *C. sakazakii* STs of isolated from infants include CC1 (79%), ST8 (14%), and ST12 (67%). The strains had been recovered from a number of sites; throat, sputum, feces, blood, and CSF.

Twenty-nine percent of *C. malonaticus* strains (*n* = 199) recorded in the database are in clonal complex 7 (**Table 1**). Strains in this complex have been isolated over the past 30 years. Strains of clinical origin had been recovered from throat, feces, and sputum samples; **Figure 1B**. It is of note that the only reported fatal neonatal meningitis cases which has been attributed to *C. malonaticus* were not from this ST. These will be discussed further below with respect to capsule profiling. The database only contained three *C. malonaticus* CC7 isolates from PIF, and there were no isolates from infant formula or milk powder manufacturing plants. This probably reflects a low incidence of this clonal complex in PIF and the manufacturing environment.

It is pertinent to emphasize that some of the predominant clinical STs were also found in the environment and this includes flies (*Musca domestica* and *Sarcophaga haemorrhoidalis*) which carry the pathovars *C. sakazakii* CC4 and ST8, as well as *C. malonaticus* ST7. Pava-Ripoll et al. (2012) reported that *Cronobacter* spp. were isolated from 14% of the flies they analyzed.

As given in **Table 1**, the remaining five species represented 18.5% of strains in the database. These 306 strains were composed of 191 STs. These species had primarily been isolated from environmental sites and are of less clinical relevance. The issue of whether only the clinically significance *Cronobacter* species need to be controlled in PIF has not been evaluated by regulatory authorities. However, the lack of epidemiological evidence of infection from all species may not be substantiated due to the frequent misidentification of *Cronobacte*r strains following the routine use of phenotyping for identification (Jackson and Forsythe, 2016).

### Diversity of *Cronobacter* Species According to Capsular Profiling

This new study enabled capsular profiling to be undertaken using genomes from across the whole *Cronobacter* genus, instead of being focussed on *C. sakazakii* and *C. malonaticus* as per the previous studies (Ogrodzki and Forsythe, 2015, 2016). Previously, the variation in the *K*-antigen and colanic acid biosynthesis encoding regions were profiled as a novel means of differentiating strains of *C. sakazakii* and *C. malonaticus* (Ogrodzki and Forsythe, 2015). The K-antigen region found was homologous to the well described K-antigen gene cluster from *E. coli* and was composed of three regions. However while *E. coli* has 60 K-antigen variants, the study of *Cronobacer* spp. by Ogrodzki and Forsythe (2015) found only two variants of the loci (designated K1 and K2). The K-antigen Region 1 (*kpsEDCS*) and Region 3 (*kpsTM*) were conserved across the two species, however there were only two variants of Region 2, designated as K1 and K2, which extended into the *kpsS* gene. The Region 2 variants differed in their length (2.28 and 3.78 kb) and CG% content (34.7 and 42.8%). They both encoded for glycosyltransferases genes for which there were no specific nearest matches (<50% similarity) in any BLAST search. Presumably the 2 variants reflect differences in the synthesized K-antigen which is exported to the cell surface, and also corresponds with the differences in the *kpsS* sequence.

This new study, using 275 genomes, shows the *K*-antigen region was present in all *Cronobacter* strains and species across the whole genus, with the exception of *C. dublinensis* ST389. Nevertheless there were no further variants have been found in this wider study compared to that of Ogrodzki and Forsythe (2015). The composition of the *K*-antigen specific capsular polysaccharide remains unknown, though it could be an important virulence or environmental fitness trait.

Previous analysis of the colanic acid biosynthesis region of *C. sakazakii* and *C. malonaticus* revealed there were two variants of the colanic acid biosynthesis gene cluster. CA2 differed from CA1 in the absence of the *galE* (encoding for UDP-N-acetyl glucosamine 4-epimerase). It is predictable that the chemical composition of the colanic acid produced by the two variants will differ, though the affect on virulence or environmental fitness trait is uncertain. The colanic acid biosynthesis region was present in all *Cronobacter* strains and species across the whole genus based on the analysis of 275 genomes. Nevertheless, as for the *K*-antigen region, there were no further variants found in this wider study. The variation is the K-antigen and colanic acid biosynthesis genes therefore do not follow the phylogeny of the genus.

*Cronobacter sakazakii* CC4 strains are associated with neonatal meningitis, and have the K2:CA2 capsule profile (Ogrodzki and Forsythe, 2015). Although this could be attributed to clonal inheritance, a limited number of non-*C. sakazakii* CC4 strains from meningitis cases have been collated from the PubMLST database. It is therefore notable that the K2:CA2 profile was also found in three other species *C. malonaticus* (*n* = 10), *C. turicensis* (*n* = 7), and *C. universalis* (*n* = 1) as well as the more distantly related *C. dublinensis* (*n* = 1); (**Table 2**). Therefore this capsule profile is neither phylogenetically nor clonaly related. In this new study, more strains of *C. malonaticus* ST60 and ST307 are shown to encode for the K2:CA2 profile. Previously only one *C. malonaticus* reported meningitis case had been documented (Ogrodzki and Forsythe, 2015). This strain (1569, ST307) was a blood isolate from a meningitis case in the United States, and was purported as being the infectious organism. This single case was taken as a possible outlier evidence for K2:CA2 profile link to meningitis. In this paper a second *C. malonaticus* K2:CA2 isolate of significance is reported. The strain Chcon-9 (ID 1494) was a CSF isolate, kindly deposited by Dr Jing-hua Cui (CDC Beijing). This second example was isolated in China (2014) from the CSF of an infant born with an EGA of 36 weeks, who had been fed fortified breast milk before developing clinical symptoms of meningitis on day 11. Strain Chcon-9 is ST60 not ST307 as per the previous *C. malonaticus* example. This latter observation further confirms the lack of congruence between the capsule profile and phylogeny. However, the remaining *C. malonaticus* ST60 strains of clinical origin also had the K2:CA2 profile and were from adults aged 26–82 years (no clinical symptoms available). The apparent correlation between capsule profile and severe infant meningitis gives a clear direction for further meningitis research with the bacterium.

None of the 55 *C. malonaticus* strains encoded for the sialic acid utilization genes (*yhch-nanKTAR*); (**Table 2**). This trait had previously been proposed as an important virulence trait in *C. sakazakii* given the occurrence of sialic acid in breast milk, mucin and gangliosides. Strains of *C. sakazakii* are able to grow on sialic acid and the ganglioside GM1 as a sole carbon source (Joseph et al., 2013b). This ability may explain the more frequent occurrence of *C. sakazakii* in severe meningitic infections compared with *C. malonaticus.*

Of the *C. turicensis* strains encoding for K2:CA2, none were clinical in origin (**Table 2**). The strains were from 5 STs which were not phylogenetically closely related (Joseph et al., 2012b). Three of these also encoded for the sialic acid utilization genes (*yhch-nanKTAR*), and a further 4 strains also encoded for these genes but had the capsule profile K1:CA2. These were the only strains outside the *C. sakazakii* species which encoded for the sialic acid utilization genes. Similar to the strains encoding for K2:CA2, the *yhch-nanKTAR* encoding strains which were identified in six different STs, clustered together according to phylogenetic analysis (Joseph et al., 2012c). The *C. turicensis* type strain lacked the sialic acid utilizing genes despite being closely related to these 6 STs. This would account for the earlier reported absence of sialic acid utilization in *C. turicensis* in laboratory studies by (Joseph et al., 2013b). Whether the acquisition of the sialic acid utilization trait by a phylogenetic cluster within *C. turicensis* is of ecological significance is uncertain given none of the strains were clinical in origin.

The K2:CA2 profile was also detected in one strain each of *C. dublinensis* (2030) and *C. universalis* (1883); (**Table 2**). Unfortunately the specific site of isolation and clinical presentation of *C. dublinensis* strain 2030 (synonym CDC 28–83) are unknown and was previously misidentified as *C. sakazakii* (Miled-Bennour et al., 2010). Currently this is the only ST301 strain in the *Cronobacter* PubMLST database, and hence the only one available for genome sequencing. The nearest related ST is ST70 for which there are 2 strains (1130 and 1462) in the database (**Figure 2**). Both strains were from Korea, one of which was from follow-up formula and the other was a food isolate (Chap et al., 2009). Neither of these strains have been sequenced to date. Whether other traits of significance occur in ST301 can be investigated in the future using comparative genomic analysis via the PubMLST *Cronobacter* database. This was considered as being outside the scope of this study. The single *C. universalis* strain (1883) encoding for K2:CA2 was isolated during a food survey in the Czech Republic (synonym DEM 3321) (Killer et al., 2015).

The occurrence of the K2:CA2 profile therefore does not follow phylogeny within the *Cronobacter* genus. Instead, there is only an association within *C. sakazakii* for severe cases of infection (NEC and meningitis) and *C. malonaticus* with severe cases of meningitis. Additionally, none of the *C. turicensis* strains encoding for K2:CA2 had been isolated from CSF. The co-occurrence of the sialic acid utilization genes did not occur in the two isolates of *C. malonaticus* isolated from meningitis cases. This observation does not discount the potential contribution of the sialic acid utilization genes in *C. sakazakii* meningitis, but does infer that it is not essential.

The *Cronobacter* genus is predicted to have evolved in the Palaeogene period of the Cenozoic era when early flowering plants evolved and coincides with the suspected natural plant habitat of the organism. The earliest branches of the genus lead to *C. dublinensis* and *C. muytjensii*, whereas *C. sakazakii is* predicted to have evolved more recently (Joseph and Forsythe, 2012; Grim et al., 2013). Possibly linked to this, **Table 1** shows the considerable genetic diversity of *C. dublinensis* and *C. muytjensii*, as reflected by the relatively large number of STs for total number of strains (107/155 and 28/57) compared with the relative smaller number of *C. sakazakii* STs (236/1126) reflecting the considerable clonality within the latter species. Such clonality results in a low amount of genomic difference between unrelated strains and therefore limits the use of conventional genotyping methods such as PFGE.

### Diversity of *Cronobacter* Species According to CRISPR-*cas* Array Profiling

CRISPR-*cas* array content has previously been strongly associated with sequence based phylogeny and hence could be used as a rapid lineage based detection method. This analysis has been applied to various *Enterobacteriaceae* (*Yersinia*, *Salmonella* and *E. coli* ) for phylogenetic, evolutionary and virulence related analysis (Makarova et al., 2015). It has also been considered as a discriminatory tool for epidemiological purposes since bacterial strains from the same geographical and temporal region should acquire the same spacers due to localized exposure to phages and plasmids, differentiating them from unrelated strains. In *Cronobacter* it was proposed that the strong clonal lineages of *C. sakazakii* resulted in a constraint in the CRISPR spacer array diversity (Ogrodzki and Forsythe, 2016). To consider if the CRISPR-*cas* array diversity varied across the genus, the arrays were determined for the less clonal species, *C. dublinensis* and *C. muytjensii* and compared with *C. sakazakii.*

Thirty *C. muytjensii* (*n* = 10) and *C. dublinensis* (*n* = 20) isolates were subject to detailed CRISPR-*cas* analysis. These two species were chosen since MLSA analysis had shown their greater diversity and more frequent novel sequence typing than the more clonal species *C. sakazakii* which has previously been analyzed. It was predicted that there would also be greater CRISPR-*cas* diversity in these two species given the probable reduction in clonal constraint of genomic recombination. The selected strains had been isolated from 11 countries over a 58 year period.

As shown in **Table 3**, both *C. dublinensis* and *C. muytjensii* included two CRISPR-*cas* operon architectures of I-E (Ecoli) and I-F (Ypseudo). The distribution of the operon types were not phylogenetically related as shown in **Figure 2**. There were a considerably high number of direct repeats and spacers in the two species, generating up to 5 CRISPR arrays and up to 66 spacers per strain compared to a maximum of 4 CRISPR arrays and 31 spacers per strain across 4 clonal *C. sakazakii* pathovars (Ogrodzki and Forsythe, 2016). Some strains in *C. dublinensis* and *C. muytjensii* lacked *cas* genes. The lack of *cas* genes was also found in the type strains of *C. universalis* and *C. condimenti* species.

Horizontal gene transfer of CRISPR and *cas* genes can occur between strains of the same species and even distant species and genera (Makarova et al., 2015). Subsequently not all strains within a species will necessarily possess the same sets of CRISPR-*cas* genes. As given in **Table 3**, within the *Cronobacter* genera there is a considerable variation in CRISPR-*cas* arrays, and even operon type and presence. Similarly, CRISPR-*cas* genes are present in enterohemorrhagic (EHEC) and Shiga toxin producing *E. coli* serotypes, but not the extra intestinal (ExPEC) *E. coli* phylogenetic group B2. Consequently, it has been proposed that the type I-E CRISPR-*cas* system could have alternative functions, such as gene expression and virulence (Touchon et al., 2011; Yin et al., 2013; García-Gutiérrez et al., 2015). Whether this relates to the variation in CRISPR-*cas* arrays in *Cronobacter* is currently unclear.

This is the first study to identify the wide-spread phylogenetic distribution of CRISPR-cas arrays across the *Cronobacter* genus, as opposed to just *C. sakazakii* (Ogrodzki and Forsythe, 2016; Zeng et al., 2017). This analysis has confirmed that differentiation within clonal lineages can be achieved using genotyping based on CRISPR-*cas* array variability.

### Genotyping across the *Cronobacter* Genus

Due to the increasing number of genome and allele sequences (MLST alleles and whole genomes) deposited in the PubMLST *Cronobacter* database, this article not only updates researchers regarding the diversity of strains within the *Cronobacter* genus as revealed by 7-loci MLST, it has also investigated the occurrence of the capsule profiles and CRISPR-*cas* arrays beyond the initial studies of *C. sakazakii* and *C. malonaticus.*

Genotyping of *Cronobacter* spp. using MLST has considerably improved our understanding of the bacterial population diversity across the genus, and led to the recognition of clonal lineages and pathovars. However, like PFGE, MLST is unable to discriminate between unrelated strains within a clonal lineage (Alsonosi et al., 2015). Therefore more discriminatory DNA-sequence based methods need to be developed. Given the increasing availability and lowering costs of NGS tools, there is an increasing trend for genome-based genotyping methods, such as CRISPR-*cas* array profiling. Such analysis may also provide additional understanding of the diversity of the species and potential virulence mechanisms.

Given the expanding nature of the *Cronobacter* PubMLST database, the figures used were as of April 2017 and may differ in precise values when accessed later. Nevertheless the general consensus of *Cronobacter* diversity will be the same. The online *Cronobacter* PubMLST database has enabled open access to considerable information on *Cronobacter* isolates which can be interrogated by researchers, industry and regulatory authorities for taxonomic, and genotyping purposes. The curation of metadata of the isolates is standardized and therefore facilitates an international contribution to collating information.

## Ethics Statement

All clinical data are taken from previous publications associated with the sequenced bacterial strains.

## Author Contributions

PO: performed the genomic analysis and collated the data. SF: Initiated the study, wrote the manuscript, and managed the project. Both authors approved the final version of the manuscript.

## Conflict of Interest Statement

The authors declare that the research was conducted in the absence of any commercial or financial relationships that could be construed as a potential conflict of interest.

## Footnotes

1 http://bigsdb.pasteur.fr/

2 http://mlst.warwick.ac.uk/

3 https://pubmlst.org

4 https://pubmlst.org/cronobacter/

5 www.pubMLST.org/cronobacter/

## Abbreviations

BIGSdb: Bacterial Isolate Genome Sequence database
*Cas*: CRISPR-associated protein-coding genes
CC: clonal complex
Cg-MLST: core genome multilocus sequence typing
CRISPR: Clustered regularly interspaced short palindromic repeats
EGA: estimated gestation age
MLST: multilocus sequence typing
NGS: next generation sequencing
PCR: polymerase chain reaction
PFGE: pulsed-field gel electrophoresis
PIF: powdered infant formula
r-MLST: ribosomal multilocus sequence typing
ST: sequence type
